# Hepatitis and Hepatitis B Virus Reactivation in Everolimus-Treated Solid Tumor Patients: A Focus on HBV-Endemic Areas

**DOI:** 10.3390/cancers16233997

**Published:** 2024-11-28

**Authors:** Chien-Hao Su, Chung-Yu Chen, Chien-Ting Liu, Yi-Hsin Yang, Pao-Chu Wu

**Affiliations:** 1 School of Pharmacy, Kaohsiung Medical University, Kaohsiung 807, Taiwan; yesorno@cgmh.org.tw (C.-H.S.); jk2975525@kmu.edu.tw (C.-Y.C.); 2 Department of Pharmacy, Chang Gung Memorial Hospital, Chiayi 613, Taiwan; 3 Division of Hematology-Oncology, Department of Internal Medicine, College of Medicine, Chang Gung University, Kaohsiung Chang Gung Memorial Hospital, Kaohsiung 833, Taiwan; m7155@cgmh.org.tw; 4 National Institute of Cancer Research, National Health Research Institutes, No. 367, Sheng-Li Rd., North District, Tainan 704, Taiwan; yihsya@gmail.com

**Keywords:** everolimus, HBV-endemic areas, hepatitis, hepatitis B reactivation, immunosuppression, solid tumors, drug induced liver injury (DILI)

## Abstract

Background: Everolimus is approved for treating breast, renal, and pancreatic neuroendocrine cancers but carries the risk of hepatitis B virus (HBV) reactivation (HBVr) and hepatitis. However, data on HBVr in everolimus-treated patients are limited. This study evaluates the risk of hepatitis and HBVr in cancer patients with current or past HBV infection. Methods: This retrospective study analyzed patients prescribed everolimus between 1 January 2011 and 31 May 2022, using a private healthcare system database in Taiwan. Patients with HBsAg positivity or HBsAg negativity and anti-HBs or anti-HBc results were included. The cumulative incidence function and risk of hepatitis from a competing risk model, which estimates Fine-Gray subdistribution hazard (SDH), were analyzed across different HBV serological subgroups. The risk of hepatitis B reactivation was also calculated. Results: Of 377 patients, 45% (36/80) of HBsAg-positive and 0.67% (2/297) of HBsAg-negative patients received nucleos(t)ide analogues (NUCs) prophylaxis. Hepatitis occurred in 28.75% of HBsAg-positive and 17.85% of HBsAg-negative patients. Baseline HBsAg positivity and exemestane use increased hepatitis risk. HBVr occurred in 11.36% (5/44) of HBsAg-positive patients without NUCs and 5.56% (2/36) with prophylaxis. Two HBsAg-negative, anti-HBc-positive patients developed severe HBVr-related hepatitis. Conclusion: Hepatitis occurred in 28.75% of HBsAg-positive and 17.85% of HBsAg-negative patients on everolimus. HBVr was common in HBsAg-positive patients but rare in HBsAg-negative individuals. HBV screening and liver function monitoring are critical for patients with past or current HBV infection receiving everolimus, especially in endemic areas.

## Simple Summary

Hepatitis B virus (HBV) infection is a significant health concern in endemic regions, particularly in Asian countries. For cancer patients with HBV, reactivation (HBVr) is a major risk due to exposure to immunosuppressants or chemotherapy, which can lead to liver failure and even death. However, HBVr is preventable with proper precautions. The aim of our retrospective study was to evaluate the risk of hepatitis and HBVr in patients with solid tumors receiving everolimus. We confirmed a substantial risk of hepatitis and HBVr associated with everolimus therapy, particularly in patients with chronic HBV infection. Our findings underscore the critical importance of thorough HBV screening and antiviral prophylaxis before initiating everolimus treatment in high-risk cancer patients.

## 1. Introduction

Hepatitis B virus (HBV) is one of the most prevalent chronic viral infections worldwide, affecting 2 billion people, with over 350 million chronic carriers [1]. The prevalence of chronic HBV infection varies by region, reaching nearly 20% in the Asia-Pacific epidemic area [1]. Although anti-HBV therapies have improved the long-term survival of chronic carriers, HBV reactivation (HBVr) significantly increases the risk of hepatic failure and mortality, particularly in patients with HBV-related cirrhosis [2]. Among oncology patients, HBVr may also lead to delays or discontinuation of systemic cancer therapy, which can adversely affect the outcomes of their underlying malignancies [3].

HBVr is not uncommon in patients receiving conventional or biologic immunosuppressive agents for autoimmune diseases. Among chronic HBV (HBsAg-positive) patients treated with biologics for rheumatological, dermatological, or gastroenterological conditions, the prevalence of HBVr is estimated to range from 16.6% to 40.5%, with only 12.5% of HBVr cases receiving antiviral prophylaxis [4]. In HBsAg-positive patients receiving non-biologic disease-modifying antirheumatic drugs (DMARDs) for inflammatory arthritis without antiviral prophylaxis, the pooled HBVr rate was 22.4% (8.1–40.8), representing a nearly 20-fold increase compared to patients with resolved HBV (HBsAg-negative and antiHBc-positive) infection (1.6%) [5]. While psoriasis patients with HBsAg positivity on biologics have a lower HBVr risk compared to those with other rheumatological conditions, this risk significantly increases to 26.6% (95% CI: 5.8–53.5%) from 4.1% (95% CI: 0.0–17.9%) if antiviral prophylaxis is needed but not administered [6]. Fortunately, HBVr-related hepatitis, liver cirrhosis, liver failure, or HBV-related deaths remain rare in patients receiving conventional and biologic immunosuppressive agents for non-oncological conditions [4,7,8].

Patients with chronic or resolved HBV infection are also at risk of viral reactivation during chemotherapy, particularly in those receiving anticancer treatment for hematologic malignancies or undergoing hematopoietic stem cell transplantation (HSCT). Data from Japan and Hong Kong indicate a 45–100% risk of HBVr and a 15% risk of hepatic failure among HBsAg-positive patients receiving HSCT without antiviral prophylaxis [8]. For patients with hematologic malignancies not on antiviral prophylaxis, the reactivation risk from anticancer therapy ranges from 25–61% among those with chronic HBV to 18% among those with past HBV infection, with similar risks reported across various subtypes [9,10,11]. Among solid tumor patients, HBVr incidence is generally lower, approximately 25% for chronic HBV cases and 3% for resolved cases, with risk varying by chemotherapy regimen and tumor subtype [3,12,13]. Patients with chronic HBV and hematologic malignancies who do not receive prophylaxis face a substantially higher risk of HBV-related liver failure (25% vs. 2%) and all-cause mortality (36% vs. 2%) compared to those with solid tumors [14]. Limited data are available on HBVr in patients receiving newer anticancer therapies, such as immunotherapy or targeted agents, with reactivation rates of 6.4–8.1% in chronic HBV and 2.1% in past HBV cases without prophylaxis [15,16,17]. HBV-related hepatitis developed in 4.3% of HBsAg-positive patients, with an HBV-related mortality rate of 18.2% (95% CI: 3.2–47.7) among those with HBVr [15,16].

Several international guidelines recommend that all patients undergo HBV screening before initiating immunosuppressive or systemic anticancer therapy, using three tests: HBsAg, anti-HBc total immunoglobulin (Ig) or IgG, and anti-HBs [8,17]. Patients with chronic HBV who are receiving any systemic anticancer therapy (e.g., cytotoxic, immunotherapy, or targeted therapy) or those at high and moderate risk of HBVr from immunosuppressive agents should receive antiviral prophylaxis throughout treatment and for a minimum of 12 months following anticancer therapy or 6 months after completing immunosuppressant therapy [8,17]. For hormonal therapy without systemic anticancer therapy, which poses minimal HBVr risk, HBV monitoring and treatment guidelines may be followed [17]. For patients with past HBV infections, the choice between antiviral prophylaxis and regular liver function monitoring depends on chemotherapy regimen, HBVr risk associated with specific immunosuppressive agents, and the presence of advanced fibrosis or cirrhosis [8,17].

Everolimus, an mTOR inhibitor with both immunosuppressive and anticancer activity, is used across multiple clinical settings: as an immunosuppressant in solid organ transplantation and as an antineoplastic agent for advanced renal cell carcinoma, metastatic hormone receptor-positive breast cancer, and pancreatic neuroendocrine tumors (P-NETs). Everolimus exerts immunosuppressive effects by promoting regulatory T-cell expansion and inhibiting T- and B-cell proliferation, which has been associated with an increased risk of infection [18]. Several cases of everolimus-related liver injury and HBVr have also been reported [19,20,21]. While most reported cases were mild and asymptomatic [6], severe and potentially fatal HBVr has been documented [22,23,24].

Despite these reports, the incidence of HBVr among everolimus users remains insufficiently characterized, making HBV screening and the decision to initiate antiviral prophylaxis challenging. Notably, while one study identifies an increased risk of everolimus-related HBVr in HBsAg-positive patients, its occurrence in patients with resolved HBV infection (HBsAg-negative, anti-HBc-positive) is less well understood [25,26]. This study aimed to determine the incidence of hepatitis and HBVr in patients treated with everolimus and to identify potential risk factors for hepatitis.

## 2. Materials and Methods

### 2.1. Study Overview

This retrospective cohort study extracted data from the Chang Gung Research Database (CGRD), the largest multi-institutional electronic medical record database, covering 6.1% of outpatients and 10.2% of hospitalized patients in Taiwan [27,28]. This database comprises clinical information of patients, including medication, intervention, and laboratory data. Disease diagnosis was made on the basis of International Classification of Diseases, Ninth Revision, Clinical Modification (ICD-9-CM) diagnostic codes before 2015 and ICD-10-CM after 2016. The comorbidities were defined by at least two outpatient diagnoses or one inpatient diagnosis in one year of everolimus initiation. The drug user was defined by at least one prescription existing during the pre-specified period. This study was reported using the Strengthening the Reporting of Observational Studies in Epidemiology (STROBE) guidelines for cohort studies [29].

### 2.2. Study Participants

**Figure 1:**
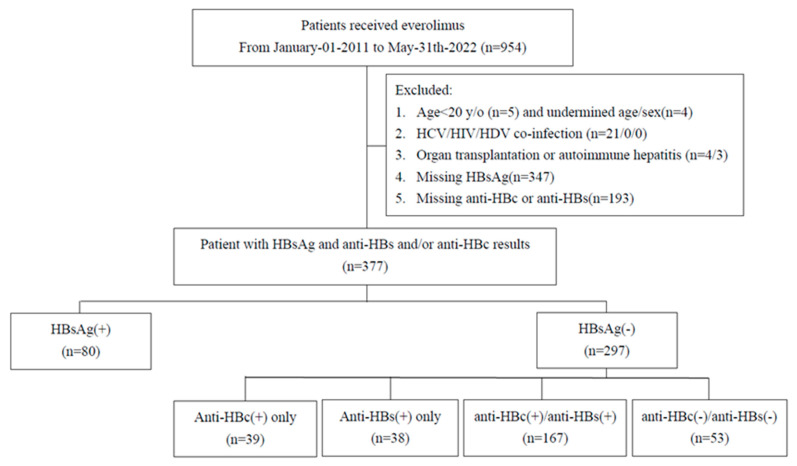
Patient flow chart.

The inclusion criteria were as follows: (1) patients who had received at least one dose everolimus therapy for cancer between 1 January 2011 and 31 May 2022. We excluded (1) Age less than 20 y/o or undermined age and sex, (2) those lacking HBV serological data (HBsAg, anti-HBc, or anti-HBs), had evidence of hepatitis C or D virus or HIV infection within five years prior to the everolimus initiation, and (3) a history of organ transplantation (ICD-9: V42.x, ICD-10: Z94.x) or autoimmune hepatitis (ICD-9: 571.49, ICD-10: K75.4) within one year of the index date. The index date was defined as the first everolimus prescription. Patient selection is depicted in Figure 1.

The patients were categorized into groups based on HBV serological status: HBsAg-positive and HBsAg-negative, with the latter divided into four subgroups (anti-HBc positive, anti-HBs positive, both anti-HBc and anti-HBs positive, and both anti-HBc and anti-HBs negative). Patient demographics (sex, age), comorbidities, and laboratory results from the year prior to the index date were collected. Nucleos(t)ide analogues (NUCs) use (lamivudine, adefovir dipivoxil, entecavir, telbivudine, tenofovir disoproxil fumarate, or tenofovir alafenamide) was defined as any NUC prescription within the year preceding everolimus initiation, regardless of current or prophylactic use. Concurrent therapies within 90 days before everolimus initiation were recorded as follows: (1) exemestane, (2) oral immunosuppressants, (3) oral steroids, (4) potential hepatotoxic agents, and (5) Traditional Chinese Medicine (TCM) prescribed by physicians. To avoid overcounting steroids used for everolimus-related stomatitis, oral prednisolone elixir and oral dexamethasone prescribed on the same day as everolimus were excluded. Serial liver biochemistry tests (AST, ALT, total bilirubin), HBV DNA, and HBsAg were retrospectively collected throughout the follow-up period, which extended from the index date until death, occurrence of hepatitis or HBVr, and/or interruption of everolimus therapy exceeding four to five half-lives (approximately 30 days) [30].

### 2.3. Main Outcome

The primary endpoint of this study was hepatitis, defined as an increase in serum alanine aminotransferase (ALT) to at least twice the upper limit of normal (ULN, i.e., 40 U/L) or twice the baseline ALT level if baseline ALT was ≥ 40 U/L [31]. Secondary endpoints included HBVr and related hepaitits. For chronic HBV infection, HBVr was defined as a ≥ 2 log (100-fold) increase in HBV DNA compared to baseline, HBV DNA ≥ 3 log (1000) IU/mL from undetectable, or HBV DNA ≥ 4 log (10,000) IU/mL if baseline HBV DNA was unavailable. In HBsAg-negative patients, HBVr was defined as either detectable HBV DNA or HBsAg seroconversion [32]. HBVr-associated hepatitis was defined as an increase in serum ALT to at least thrice-fold ULN or thrice the baseline ALT level if baseline ALT was ≥40 U/L and concurrent HBVr [25]. If ALT increased to at least ten-fold ULN or baseline or to at least thrice ULN or baseline AND bilirubin at least twice ULN or INR over 1.5 and concurrent HBVr, this was considered as HBVr-associated severe hepatitis [25]. In HBVr subgroups, we persistently follow up to trace the survival status to until the last visit in hospitals or the conclusion of the follow-up period (31 May 2023).

### 2.4. Statistical Analysis

Descriptive statistics were employed to summarize continuous variables (mean ± SD or median with interquartile range (IQR) and categorical variables (frequency and percentage). The baseline characteristic differences were compared across five subgroups by conducting an Analysis of Variance (ANOVA) for numeric variables and Pearson’s chi-square tests or Fisher’s exact test for categorical variables. The cumulative incidence of hepatitis was calculated and compared using the Fine–Gray method, considering death as a competing risk for the outcomes of treatment events. To control for multiple comparisons and reduce the risk of type I errors, we applied the Benjamini–Hochberg False Discovery Rate (FDR) method, with a significance threshold of *p* < 0.05. The Fine–Gray regression model allows for the assessment of hepatitis risk and was used to derive the subdistribution hazard ratios (SHRs) with 95% CI of the hepatitis risk in current and past HBV infection patients. The Fine–Gray model was chosen to estimate hepatitis risk in the setting of the competing mortality risk because it is the most appropriate model to assess the prognostic effect of the intervention and the individual risk, compared with other competing risk models such as the cause-specific Cox regression model, which is most appropriate for etiology studies. A two-sided *p*-value < 0.05 was considered statistically significant. Data management and statistical analyses were performed using SAS (version 9.4) and R software (version 4.0.0).

## 3. Results

### 3.1. Clinical Characteristics of Patients in the Current Study

A total of 954 patients initiating everolimus between 1 January 2011 and 31 May 2022, were identified. After excluding patients with missing HBV serological data, 377 patients (80 HBsAg-positive, 297 HBsAg-negative) were included for analysis with a median follow-up of 3.8 months (IQR 2.47, 7.07). Among HBsAg-negative patients, 39 were anti-HBc positive only, 38 were anti-HBs positive only, 167 were both anti-HBc and anti-HBs positive, and 53 were negative for both (shown in Figure 1).

The mean age at everolimus initiation was 58.89 years, with 80.11% of patients being female. The primary indications for everolimus were breast cancer (*n* = 238, 63.13%), renal cell carcinoma (*n* = 47, 12.47%), and neuroendocrine tumor or carcinoid (*n* = 31, 8.22%). Additionally, 64 patients (16.98%) had secondary liver malignancies. Over 90% had no history of cirrhosis or hepatocellular carcinoma, and 80% presented with normal baseline ALT levels. Twenty-one percent of patients had been exposed to potential hepatotoxic agents, and seven percent to TCM, within 90 days before initiating everolimus.

In the HBsAg-positive cohort, 36 patients (45%) were on NUC treatment at baseline, with an additional 5 patients (6.25%) started NUC therapy during everolimus treatment. Antiviral NUC use was rare in the HBsAg-negative cohort (*n* = 2, 0.67%). Baseline characteristics were generally similar across the subgroups, except for age, baseline cholesterol, and NUC use at baseline or during everolimus treatment. Detailed patient characteristics are provided in Table 1.

### 3.2. Primary Outcome: Hepatitis During On-Everolimus Treatment

A total of 76 patients (20.16%) experienced hepatitis, predominantly in the HBsAg-positive group (28.75%). Severe hepatitis (ALT > 5× ULN) occurred in 5.84% of patients, with ALT > 10× ULN in 3.18% and icteric flare in 2.65%. The median time to hepatitis was 2.32 months (IQR: 1.28, 4.1). The detailed results of hepatitis in patients with HBV virological subgroup are shown in Table 2.

**Figure 2:**
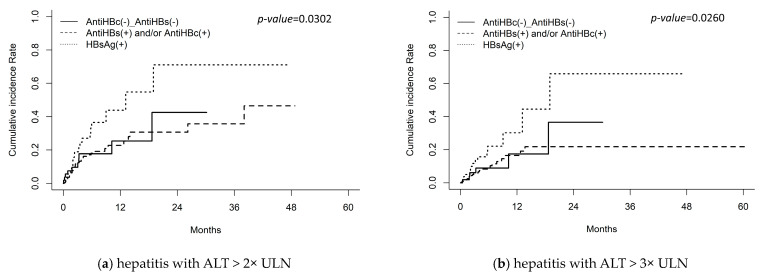

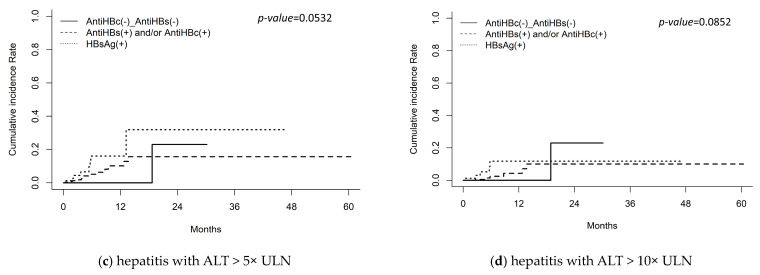
Competing risk analysis for (**a**) hepatitis flare with ALT > 2× ULN. Adjust *p*-values for: HBsAg(+) vs. AntiHBs(+) and/or AntiHBc(+): *p* = 0.026; HBsAg(+) vs. AntiHBc(−)_AntiHBs(−): *p* = 0.160; AntiHBc(−)_AntiHBs(−) vs. AntiHBs(+) and/or AntiHBc(+): *p* = 0.7396 (**b**) hepatitis with ALT > 3× ULN, Adjust *p*-values for: HBsAg(+) vs. AntiHBs(+) and/or AntiHBc(+): *p* = 0.024; HBsAg(+) vs. AntiHBc(−)_AntiHBs(−): *p*= 0.172; AntiHBc(−)_AntiHBs(−) vs. AntiHBs(+) and/or AntiHBc(+): *p* = 0.743 (**c**) hepatitis flare with ALT > 5× ULN, and (**d**) hepatitis flare with ALT > 10× ULN in patients with positive hepatitis B surface antigen (HBsAg), patients with negative HBsAg, whereas positive antibody to HBsAg (anti-HBs) and/or antibody to hepatitis B core antigen (anti-HBc), and patients with negative HBsAg, anti-HBs and anti-HBc. Abbreviations: ALT, alanine aminotransferase; ULN, upper limit of normal.

Cumulative incidence analysis revealed a significantly higher risk of hepatitis in the HBsAg-positive group compared to the HBsAg-negative group (*p* = 0.030). A significant difference in hepatitis was observed between the HBsAg-positive group and the anti-HBs-positive and/or anti-HBc-positive group (*p* = 0.026), but not between other subgroup comparisons (Figure 2).

Univariable analysis identified HBsAg positivity, secondary liver metastasis, and concurrent exemestane use as risk factors for hepatitis. Multivariable analysis confirmed HBsAg positivity (SHR: 2.25, 95% CI: 1.36–3.75) and concurrent exemestane use (SHR: 2.32, 95% CI: 1.22–4.40) as independent risk factors, while younger age demonstrated a protective effect (SHR: 0.47, 95% CI: 0.27–0.81) (Table 3).

In the HBsAg-positive subgroup, younger age (SHR: 0.14, 95% CI: 0.02–0.75) and baseline NUC use (SHR: 0.23, 95% CI: 0.08–0.64) were associated with a lower risk of hepatitis. Conversely, in the HBsAg-negative subgroup, liver metastasis (SHR: 2.32, 95% CI: 1.08–4.98) and concurrent exemestane use (SHR: 2.86, 95% CI: 1.20–6.78) were linked to an increased risk of hepatitis (Table 3).

### 3.3. Secondary Outcome: HBVr On-Everolimus Treatment

Among the 80 HBsAg-positive patients on everolimus, a total of 7 patients experiences HBVr, of whom 2 patients were receiving baseline NUCs prophylaxis, with a median interval time of 5.67 months (IQR: 3.23, 9.10) until reactivation. The majority were female (*n* = 6, 85.71%) and the primary indication for everolimus use was breast cancer (85.71%). No immunosuppressant or steroid exposure was detected 90 days before everolimus initiation. The incidence of HBV reactivation was 5.56% and 11.36% in patients with and without antiviral NUC prophylaxis, respectively.

Among the HBVr cases, 57% developed HBVr-related hepatitis, with 75% classified as severe. The median peak serum ALT level was 1448 IU/mL (range: 313–2217 IU/mL). None of the HBsAg-positive patients experienced HBV seroclearance during everolimus treatment. Two patients died 190 and 268 days after HBVr, respectively (Table 4).

In HBsAg-negative patients with anti-HBc positivity, two cases developed severe HBVr-related hepatitis, with a median peak serum ALT level of 465 IU/mL (range: 432–498 IU/mL). The median time to HBV seroconversion was 8.95 months (range: 4.97–12.93). One case resulted in death 557 days after HBVr (Table 4).

## 4. Discussion

This cohort study investigated the association between everolimus use and hepatitis, and HBVr, in patients with current or past HBV infection. Hepatitis occurred in 28.75% of HBsAg-positive and 17.85% of HBsAg-negative patients, with most cases being mild, and icteric flare was uncommon. HBVr was observed in 11.36% of HBsAg-positive patients without NUCs prophylaxis and HBV seroconversion occurred in less than 1% of patients with past HBV infection. The probability of HBsAg seroclearance was also low (0%) in the subgroup of patients with HBsAg positivity.

Liver test abnormalities in cancer patients can have multiple etiologies, with liver metastasis, drug-induced liver toxicity (DILI), pancreaticobiliary pathology, and chronic viral hepatitis being the four most common causes [33]. The impact of liver metastasis on immune-related hepatitis remains controversial in patients treated with immune checkpoint inhibitors [34,35]. Everolimus, an immunomodulator with extensive hepatic metabolism, may cause liver injury either directly or through toxic metabolic intermediates, possibly due to its high lipophilicity (log P of 5.01) [19]. Additionally, mTOR inhibitors can modulate the immune response in a context-dependent manner, potentially increasing susceptibility to opportunistic infections or reactivation of latent infections [36,37]. Although previous studies on everolimus have reported variable rates of hepatitis, the incidence of all-grade and high-grade (grade 3–4, ALT > 5 ULT) ALT elevation has ranged from 11% to 47% and 1.5% to 4%, respectively, with no clear association with tumor type, chemotherapy regimen, or combination therapy [19].

Several cancer chemotherapies, immunosuppressive therapies, biologic treatments, and solid organ or bone marrow transplants have been associated with HBVr and related hepatitis. In Taiwan, where national health insurance covers over 99% of the population, everolimus is reimbursed for metastatic breast cancer and P-NETs, although it is generally not combined with chemotherapy or other targeted therapies for these indications. Everolimus may be used alone or with lenvatinib, which has been linked to hepatitis in RCC therapy [38,39]. However, lenvatinib is self-paid in Taiwan and is rarely co-prescribed with everolimus for RCC, accounting for only 12.46% of indications in this study. After controlling for the potential effects of concurrent chemotherapy or targeted therapy and adjusting for common hepatotoxic medications (immunosuppressants, oral steroids, potentially hepatotoxic agents, and TCM exposure) and comorbidities (such as liver metastasis), our findings underscore the significance of HBsAg status and concomitant drug use (such as exemestane) as risk factors for hepatitis in patients receiving everolimus for solid tumors.

Despite recommendations for HBV screening and antiviral NUC prophylaxis before starting immunosuppressive therapy, suboptimal screening rates and insufficient antiviral prophylaxis remain significant issues in clinical practice. A previous meta-analysis of randomized controlled trials (RCTs) reported pre-chemotherapy HBV screening rates around 57% (46–68%), with variations depending on the type of chemotherapy, HBV endemicity, and whether the malignancies were solid or hematologic in nature [40]. This gap is critical, given the well-established link between chemotherapy and HBVr risk [40]. According to a post-hoc analysis of an RCT, everolimus-related HBVr is currently considered high-risk, with HBVr-associated hepatitis occurring in 4 out of 26 (15.38%) HBsAg-positive patients who did not receive NUC prophylaxis [30]. Additionally, several case reports suggest that everolimus-related HBVr typically occurs between 0.5 to 6 months after starting everolimus, with fatal outcomes in 3 of 5 cases [22]. Antiviral NUC prophylaxis has been shown to reduce the risk of chemotherapy-related HBVr, HBVr-associated hepatitis, and interruptions in cancer treatment among patients with chronic HBV infection.

In the current study, the screening rate for HBsAg screening rate was 39.1%, comparable to previous reports [40], and only half of the HBsAg-positive patients received NUC prophylaxis. Our findings confirm the high risk of HBVr (>10%) in HBsAg-positive patients on everolimus without NUC prophylaxis, with a median time to HBVr of 5.67 months (IQR: 3.23–9.10), consistent with prior studies. Our study also demonstrated that prophylactic NUC use significantly reduced the risk of everolimus-associated hepatitis in HBsAg-positive patients, likely due to its ability to inhibit HBV replication and reactivation. However, despite prophylactic antiviral therapy, HBV reactivation occurred in two HBsAg-positive cases. Similar findings have been reported in studies focused on chemotherapy with immunomodulators [41,42,43], which may be attributed to the development of resistance to antiviral therapy from prior exposure or irregular adherence to antiviral treatment, such as missed doses for unavoidable reasons [41,44]. These results further underscore the critical importance of comprehensive HBV screening and consistent NUC prophylaxis before initiating everolimus in clinical practice.

The incidences of HBsAg seroreversion and hepatitis in patients with prior HBV exposure remain limited and unclear. Observational studies have reported varying risks of HBVr in chronic hepatitis B patients receiving newer immunosuppressants, with rates ranging from 1% to 14% across different studies [2]. HBVr in solid tumor patients with resolved HBV infection is relatively rare; however, a low anti-HBs antibody titer (<10.0 mIU/mL) and high average daily dexamethasone dose (>1.0 mg/day) have been reported as significant risk factors [45]. In our study, 2 out of 204 (0.98%) resolved HBV cases developed HBVr during everolimus treatment. Notably, secondary liver malignancies and concurrent exemestane use were associated with an increased risk of hepatitis, while the absence of anti-HBs antibodies showed a trend, though not statistically significant, toward increased HBVr risk, which may be due to small sample size and missing antiHBs titer data.

Previous studies indicate that liver metastasis, alone or combined with other factors (mixed etiology), is the most common cause of liver test abnormalities in cancer patients receiving chemotherapy [33]. Among chronic HBV patients, those with secondary liver malignancies also face a higher risk of HBVr, although this phenomenon is rarely reported in patients with resolved HBV infection, warranting further research to clarify its role [13,46]. Exemestane, widely used as adjuvant endocrine therapy for postmenopausal women with hormone receptor-positive breast cancer, has been associated with hepatotoxicity in some case reports [47,48]. Like everolimus, exemestane is metabolized in the liver and produces multiple metabolites, though the specific mechanism behind its hepatotoxicity remains unclear. Clinicians should be aware of the potential additive hepatotoxicity when combining everolimus with exemestane.

### 4.1. Limitations of the Study

Several limitations inherent to this retrospective study should be considered. First, the study’s retrospective design and reliance on electronic medical records may have introduced selection bias and missing data, particularly regarding HBV serological markers and liver function tests. Cases of isolated anti-HBs positivity might represent HBV vaccine recipients; however, we were unable to clearly identify these due to incomplete anti-HBc data. Excluding patients without both anti-HBs and anti-HBc results may have introduced negative bias, as previous vaccine recipients were included, potentially underestimating the risk of hepatitis flare.

Second, fluctuations in ALT levels might not be solely related to HBVr but could also be attributed to other factors, such as DILI, hepatic steatosis, or systemic infections. Additionally, patients often receive multiple drugs, making it challenging to isolate the specific role of everolimus in increasing the risk of liver injury and HBVr in a real-world setting. Incomplete data on concurrent medications and other potential confounders may have also impacted our findings.

Third, the irregular intervals of ALT monitoring, combined with the fact that HBV serological markers and HBV DNA viral load were typically only checked when ALT levels were elevated during everolimus treatment, could affect the accuracy of hepatitis timing and potentially lead to an underestimation of the HBVr risk associated with everolimus.

Despite these limitations, this study is the first and largest to systematically assess the impact of current or past HBV infection on hepatitis and HBVr emergence associated with everolimus. We applied stringent exclusion criteria and utilized multivariable analysis to minimize potential biases. Our study provides valuable insights into the hepatotoxic risks of everolimus in patients with HBV infection; however, further prospective studies are warranted.

### 4.2. The Strength of the Study

The strength of this study lies in several key areas:

1. Comprehensive HBV Risk Assessment: It provides valuable insights into the risks of hepatitis and HBV reactivation (HBVr) in patients treated with everolimus, a relatively underexplored area, particularly for those with solid tumors. This fills an important knowledge gap in the context of immunosuppressive therapies.
2. Real-World Data: The study utilizes a large dataset from a private healthcare system, offering real-world evidence on the incidence of hepatitis and HBVr, including the impact of prophylactic antiviral therapy in high-risk populations.
3. Focus on High-Risk Populations: It specifically addresses cancer patients with current or past HBV infection, especially in HBV-endemic regions like Taiwan, where this issue is particularly relevant. This makes the findings highly applicable to similar clinical settings worldwide.
4. Clear Implications for Clinical Practice: The study highlights the importance of HBV screening, prophylaxis, and monitoring in cancer patients receiving everolimus, reinforcing guidelines and potentially influencing treatment protocols in high-risk populations.
5. Detailed Outcome Analysis: The study’s detailed analysis of HBVr-related hepatitis severity, ALT levels, and patient outcomes adds depth to the understanding of how everolimus impacts liver health, especially in HBsAg-positive individuals.

## 5. Conclusions

In conclusion, hepatitis occurs in approximately 28.75% of HBsAg-positive and 17.85% of HBsAg-negative patients receiving everolimus, with most cases being mild and severe hepatitis or icteric flare remaining uncommon. Both current or past HBV infection and concurrent exemestane exposure influence the risk of hepatitis associated with everolimus. However, HBV screening rates remain inadequate in solid cancer patients initiating everolimus.

Our findings underscore the critical importance of comprehensive HBV screening, assessment of secondary liver malignancy, evaluation of exemestane exposure, and antiviral prophylaxis before starting everolimus in high-risk populations. While this study offers valuable insights, its retrospective design and data limitations call for further prospective studies to clarify underlying mechanisms and guide optimal management strategies. By implementing rigorous screening and preventive measures, healthcare providers can mitigate the hepatotoxic risks of everolimus and improve patient outcomes.

## Figures and Tables

**Table 1:** Clinical characteristics of patients at the time of starting everolimus treatment.

	HBsAg(+) (*n* = 80)	HBsAg Negative (*n* = 297)				
		Anti-HBc(+) (*n* = 39)	Anti-HBs(+) (*n* = 38)	Anti-HBc(+)/ Anti-HBs(+) (*n* = 167)	Anti-HBc(−)/ Anti-HBs(−) (*n* = 53)	*p* Value
Age (yr), mean (SD)	56.5 (9.97)	62.62 (10.90)	52.98 (15.10)	60.9 (11.76)	57.69 (11.73)	0.0002
Male gender, *n* (%)	20 (25%)	10 (26.32%)	3 (7.69%)	31 (18.56%)	11 (20.75%)	0.2008
Breast cancer, *n* (%)	43 (53.75%)	24 (61.54%)	26 (68.42%)	115 (68.86%)	30 (56.60%)	0.1440
Prior CDK 4/6 inhibitor in 1 year, *n* (%)	4 (5%)	2 (5.26%)	6 (15.38%)	7 (4.19%)	7 (13.21%)	0.1438
Concurrent drug exposure, *n* (%) Exemestane Potential hepatoxic agents ^#1^ Traditional Chinese Medicine	37 (46.25%) 19 (23.75%) 11 (13.75%)	22 (57.86%) 7 (18.42%) 2 (5.26%)	22 (56.41%) 11 (28.21%) 3 (7.69%)	106 (63.47%) 29 (17.26%) 10 (5.95%)	28 (52.83%) 16 (30.19%) 3 (5.66%)	0.1603 0.2306 0.2408
Concurrent immunosuppressant ^#2^, *n* (%) ≧28 days <28 days	4 (5%) 2 (2.5%) 2 (2.5%)	3 (7.89%) 2 (5.26%) 1 (2.63%)	1 (2.56%) 0 (0%) 1 (2.56%)	7 (4.17%) 6 (3.57%) 1 (0.60%)	1 (1.89%) 1 (1.89%) 0 (0%)	0.6241
Concurrent steroid exposure, *n* (%) ≧20 mg/day <20 mg/day	9 (11.25%) 2 (2.50%) 7 (8.75%)	5 (13.16%) 4 (10.53%) 1 (2.63%)	2 (5.13%) 1 (2.56%) 1 (2.56%)	20 (11.90%) 4 (2.38%) 16 (9.52%)	8 (15.09%) 1 (1.89%) 7 (13.21%)	0.6674
Cirrhosis, *n* (%)	6 (7.5%)	3 (7.89%)	2 (5.13%)	2 (1.20%)	4 (7.55%)	0.0239
Hepatocellular carcinoma, *n* (%)	6 (7.50%)	2 (5.26%)	2 (5.13%)	6 (3.59%)	2 (3.77%)	0.7027
Liver metastasis, *n* (%)	17 (21.25%)	5 (13.16%)	8 (20.51%)	24 (14.29%)	10 (18.87%)	0.5955
Dyslipidemia, *n* (%)	11 (20%)	4 (25%)	2 (20%)	15 (28.30%)	6 (46.15%)	0.6279
Alcohol related illness, *n* (%)	0 (0%)	1 (2.63%)	0 (0%)	1 (0.6%)	0 (0%)	0.3976
Creatinine(mg/dL), mean (SD)	0.88 (0.65)	1.05 (0.87)	0.8 (0.58)	0.98 (1.11)	1.04 (1.59)	0.2819
Cholesterol (mg/dL), mean (SD)	169.69 (16.78)	216.2 (38.00)	227.60 (60.90)	171.56 (36.67)	184 (38.98)	0.0482
Triglyceride (mg/dL), mean (SD)	133.83 (89.94)	205.30 (180.01)	152.50 (81.30)	139.27 (67.53)	163.8 (126.95)	0.5983
HbA1c (%), mean (SD)	6.66 (1.50)	7.84 (2.40)	5.88 (0.61)	6.71 (1.56)	6.02 (0.89)	0.1960
Platelet (×103/µL), mean (SD)	251.05 (122.10)	211.89 (113.92)	219.33 (94.16)	232.67 (83.65)	252.11 (104.77)	0.2860
INR, mean (SD)	1.07 (0.10)	1.10 (0.14)	1.06 (0.12)	1.08 (0.14)	1.05 (0.15)	0.4925
Albimin (mg/dL), mean (SD)	3.71 (0.70)	3.53 (0.71)	3.60 (0.63)	3.74 (0.70)	3.72 (0.69)	0.5480
Total bilirubin (mg/dL), mean (SD)	0.56 (0.27)	0.82 (0.92)	0.56 (0.46)	0.68 (1.04)	0.57 (0.65)	0.2378
ALT (U/L), median (IQR)	22 (15, 34)	24 (16.5, 38.5)	19 (14, 41)	18 (18, 31)	25.5 (17, 40)	0.1111
AST (U/L), median (IQR)	28.5 (23, 37.5)	29 (21, 37)	26 (23, 34.5)	27 (22, 39)	29 (23.5, 39.5)	0.9096
Alkaline phosphatase (U/L), mean (SD)	104.51 (64.27)	111.37 (110.76)	109.61 (114.52)	109.83 (126.58)	115.35 (115.54)	0.7443
HBV DNA (log10 IU/mL) Undetectable/low (<2000), *n* (%) No measurement, *n* (%)	3.1 (1.72, 4.28) 25 (31.25%) 38 (47.50%)	- 5 (13.16%) 33 (86.84%)	- 1 (2.56%) 38 (97.44%)	- 9 (5.39%) 158 (94.61%)	- 1 (1.89%) 52 (98.11%)	- 0.0704 0.0242
NUC At baseline, *n* (%) NUC Newly started during Rx, *n* (%)	36 (45%) 5 (6.25%)	1 (2.63%) 0 (0%)	0 (0%) 0 (0%)	1 (0.6%) 0 (0%)	0 (0%) 0 (0%)	<0.0001

#1 Potential hepatoxic agents included antituberculosis agents (isoniazid, rifampin, pyrazinamide), antimicrobials (amoxicillin-clavulanic acid, nitrofurantoin, cotrimoxazole, minocycline, cefazolin, azithromycin, ciprofloxacin, levofloxacin, flucloxacillin, terbinafine) and others (diclofenac, nimesulide, ibuprofen, cyproterone, carbamazepine, methyldopa, atorvastatin, flutamide, ticlopidine, nivolumab, ipilimumab, infliximab, disulfitram, metamizole, ribociclib, azathioprine, methotrexate). #2 Immunosupressants included oral azathioprine, cyclosporin, mycophenolate, sirolimus, tacrolimus, hydroxychloroquine, sulfasalazine, methotrexate, cyclophosphamide, and leflunomide. Abbreviations: SD: standard deviation; IQR: interquartile range; INR: international normalized ratio; ALT: alanine aminotransferase; AST: aspartate aminotransferase; HBV: hepatitis B virus; NUC: nucleos(t)ide analogues, HBsAg: hepatitis B surface antigen.

**Table 2:** Hepatitis after everolimus initiation in different serological groups.

	HbsAg Positive (*n* = 80)	HbsAg Negative			
		Anti-HBc(+) Only (*n* = 37)	Anti-HBs(+) Only (*n* = 37)	Anti-HBc(+)/ Anti-HBs(+) (*n* = 167)	Anti-HBc(−)/ Anti-HBs(−) (*n* = 53)
Follow up (months), median (IQR)	3.57 (1.95, 6.28)	3.57 (2.47, 7.50)	3.63 (2.50, 7.10)	4.10 (2.87, 8.00)	3.80 (2.40, 7.07)
Hepatitis, *n* (%) ^a^ ALT > 2× ULN ALT > 3× ULN ALT > 5× ULN ALT > 10× ULN	23 (28.75%) 15 (18.75%) 8 (10%) 5 (6.25%)	8 (21.05%) 7 (18.42%) 4 (10.53%) 2 (5.26%)	6 (15.38%) 4 (10.26%) 1 (2.56%) 0 (0%)	29 (17.37%) 13 (7.78%) 8 (4.79%) 4 (2.40%)	10 (18.87%) 6 (11.32%) 1 (1.89%) 1 (1.89%)
Time to hepatitis (months), median (IQR) ALT > 2× ULN ALT > 3× ULN ALT > 5× ULN ALT > 10× ULN	2.30 (1.57, 5.63) 2.63 (1.00, 5.77) 4.52 (2.07, 5.70) 3.67 (2.73, 5.63)	2.32 (1.70, 5.25) 2.10 (1.30, 6.30) 3.38 (1.47, 8.47) 8.7 (4.67, 12.73)	2.80 (1.43, 3.37) 2.80 (2.12, 5.25) 1.43 (1.43, 1.43) -	2.07 (1.27, 4.00) 3.77 (1.87, 6.70) 6.40 (3.88, 9.08) 7.27 (4.20, 11.22)	2.43 (0.43, 3.30) 2.62 (1.87, 10.20) 18.67 (18.67, 18.67) 18.90 (18.90, 18.90)
Iteric hepatitis ^b^	3 (3.75%)	2 (5.26%)	0 (0%)	3 (1.8%)	2 (2%)
Coagulopathy (INR > 1.7)	4 (5%)	1 (2.7%)	2 (5.4%)	7 (4.19%)	2 (3.77%)

^a^ Hepatitis was defined as serum ALT raised above 2 times, 3 times, 5 times, or 10 times of ULN (40 U/L) after receiving everolimus if the baseline ALT was normal. If baseline ALT > 40 U/L, it would be 2 times, 3 times, 5 times, or 10 times of the baseline. ^b^ Icteric flare was defined as ALT raised > 3× ULN (or baseline) together with serum total bilirubin > 2× ULN (or baseline). Abbreviations: IQR: interquartile range; ALT: alanine aminotransferase; ULN: upper limited normal; INR: international normalized ratio.

**Table 3:** Predictors of on-treatment hepatic during everolimus treatment.

	Univariate Analysis		Multivariable Analysis	
	SHR (95% CI)	*p*	SHR (95% CI)	*p*
All patients				
Age (yr) (≥50 vs. <50)	0.623 (0.383–1.013)	0.0564	0.472 (0.274–0.814)	0.0069
Dyslipidemia (yes vs. no)	0.624 (0.249–1.566)	0.3154		
Gender (Male vs. Female)	0.561 (0.280–1.126)	0.1040	0.856 (0.334–2.195)	0.7463
Baseline ALT (>40 vs. ≦40 U/L)	1.376 (0.799–2.369)	0.2503	1.205 (0.670–2.167)	0.5330
HBsAg (+) vs. HBsAg (−)	1.918 (1.188–3.097)	0.0077	2.254 (1.355–3.748)	0.0017
Cirrhosis (yes vs. no)	1.125 (0.329–3.850)	0.8515	1.058 (0.252–4.435)	0.9384
Liver metastasis (yes vs. no)	1.862 (1.112–3.118)	0.0181	1.750 (0.990–3.092)	0.0541
Concurrent medication exposure Exemestane (yes vs. no) Immunosuppressant (yes vs. no) Steroid (yes vs. no) Potential hepatoxic agents (yes vs. no) Traditional Chinese Medicine (yes vs. no)	1.825 (1.099–3.032) 1.433 (0.524–3.918) 0.977 (0.463–2.064) 1.051 (0.608–1.814) 1.097 (0.471–2.555)	0.0202 0.4828 0.9515 0.8594 0.8308	2.318 (1.221–4.399) 1.348 (0.557–3.262) 0.880 (0.400–1.939) 0.955 (0.533–1.711) 0.999 (0.421–2.372)	0.0101 0.5085 0.7520 0.8769 0.9985
HBsAg-positive patients				
Age (yr) (≥50 vs. <50)	0.407 (0.162–1.021)	0.0555	0.136 (0.024–0.754)	0.0224
Gender (Male vs. female)	1.034 (0.390–2.746)	0.9460	0.609 (0.106–3.506)	0.5788
Baseline ALT (>40 vs. ≦40 U/L)	1.022 (0.382–2.733)	0.9660	0.761 (0.244–2.370)	0.6369
No prevention NUC use at baseline NUC newly started during Tx	Ref 0.411 (0.166–1.021) 0.618 (0.200–1.907)	0.0555 0.4025	0.229 (0.081–0.642) 0.342 (0.110–1.058)	0.0051 0.0626
Liver metastasis (yes vs. no)	1.032 (0.424–2.512)	0.9439	2.132 (0.833–5.455)	0.1144
Concurrent medication exposure Exemestane (yes vs. no) Immunosuppressant (yes vs. no) Steroid (yes vs. no) Potential hepatoxic agents (yes vs. no) Traditional Chinese Medicine (yes vs. no)	1.432 (0.658–3.116) 3.496 (0.871–14.035) 1.792 (0.526–6.099) 1.087 (0.357–3.312) 0.973 (0.270–3.514)	0.3651 0.0775 0.3506 0.8830 0.9671	2.332 (0.919–5.916) 1.699 (0.287–10.064) 1.145 (0.336–3.896) 0.582 (0.151–2.243) 1.277 (0.327–4.978)	0.0746 0.5592 0.8286 0.4317 0.7250
HBsAg-negative patients				
Age (yr) (≥50 vs. <50)	0.681 (0.382–1.211)	0.1906	0.618 (0.335–1.140)	0.1235
Gender (Male vs. female)	0.356 (0.129–0.983)	0.0462	0.713 (0.200–2.534)	0.6006
Baseline ALT (>40 vs. ≦40 U/L)	1.548 (0.821–2.919)	0.1764	1.299 (0.642–2.630)	0.4676
Anti-HBc(−)/anti-HBs(−) Anti-HBs ≧ 10 U/mL Anti-HBs < 10 U/mL or no available	ref 0.651 (0.311–1.364) 1.596 (0.749–3.399)	0.2557 0.2257	0.850 (0.374–1.930) 2.284 (0.924–5.649)	0.6968 0.0737
Cirrhosis (yes vs. no)	2.624 (0.741–9.292)	0.1349	2.115 (0.529–8.448)	0.2892
Liver metastasis (yes vs. no)	2.222 (1.202–4.105)	0.0108	2.324 (1.084–4.983)	0.0303
Concurrent medication exposure Exemestane (yes vs. no) Immunosuppressant (yes vs. no) Steroid (yes vs. no) Potential hepatoxic agents (yes vs. no) Traditional Chinese Medicine (yes vs. no)	2.471 (1.246–4.904) 0.989 (0.249–3.927) 0.820 (0.323–2.080) 1.110 (0.590–2.088) 0.973 (0.270–3.514)	0.0097 0.9871 0.6754 0.7465 0.9671	2.856 (1.204–6.776) 0.599 (0.113–3.181) 0.844 (0.305–2.331) 1.139 (0.581–2.230) 0.653 (0.157–2.710)	0.0172 0.5476 0.7433 0.7051 0.5571

Abbreviations: NUC: nucleos(t)ide analogue; HBsAg: hepatitis B surface antigen; anti-HBc: antibody to hepatitis B core antigen; anti-HBs: antibody to HBsAg.

**Table 4:** HBV reactivation after everolimus in patients subgroup with HbsAg(+) and HbsAg(−)/Anti-HBc(+).

	HbsAg(+) (*n* = 7)	HbsAg(−)/Anti-HBc(+) (*n* = 2)
Age ≥ 50 y/o, *n* (%)	5 (71.43%)	2 (100%)
Female, *n* (%)	6 (85.71%)	2 (100%)
Everolimus indication, *n* (%) Breast cancer Neuroendocrine tumor/Carcinoid	6 (85.71%) 1 (14.29%)	2 (100%) 0 (0%)
Concurrent exemestane use, *n* (%)	4 (57.14%)	2 (100%)
Concurrent immunosuppressant, *n* (%)	0 (0%)	0 (0%)
Concurrent steroid use, *n* (%)	0 (0%)	0 (0%)
NUC at baseline, *n* (%)	2 (28.57%)	1 (50%)
HBV viral reactivation, *n* (%) HBV DNA ≧ 10,000 IU/mL if baseline not available HBV DNA ≧ 1000 IU/mL from previously undectable ≧2 log increase in HBV DNA from baseline	3 (42.86%) 1 (14.29%) 3 (42.86%)	1 (50%) 1 (50%) 0 (0%)
Time to HBVr (months), median (IQR)	5.67 (3.23, 9.10)	8.95 (4.97, 12.93)
HBVr related hepatitis (ALT > 3× ULN), *n* (%) Median (Range)	4 (57.14%) 1448 (313–2217)	2 (100%) 465 (432–498)
HBVr related severe hepatitis, *n* (%) ALT > 10 UNN from baseline ALT > 3× ULN and INR > 1.5 or bilirubin > 2× ULN	3 (42.86%) 3 (42.86%) 2 (28.57%)	2 (100%) 2 (100%) 2 (100%)
HBVr related decompensation, *n* (%) INR ≧ 3 Bilirubin ≧ 2	3 (42.86%) 2 (28.57%) 3 (42.86%)	2 (100%) 0 (0%) 2 (100%)
HBsAg seroclearance, *n* (%)	0 (0%)	-
HBsAg seroconversion, *n* (%)	-	2 (100%)
Death	2 (190 days, 268 days)	1 (557 days)

Abbreviations: NUC: nucleos(t)ide analogue; ULN, upper limit of normal; HBV: hepatitis B virus; INR: international normalized ratio.

## Data Availability

The data that support the findings of this study are available from the corresponding author upon reasonable request.
